# The Omics Dashboard for Interactive Exploration of Metabolomics and Multi-Omics Data

**DOI:** 10.3390/metabo14010065

**Published:** 2024-01-19

**Authors:** Suzanne Paley, Peter D. Karp

**Affiliations:** Bioinformatics Research Group, SRI International, Menlo Park, CA 94025, USA; suzanne.paley@sri.com

**Keywords:** metabolomics, multi-omics, systems biology, data visualization

## Abstract

The Omics Dashboard is a software tool for interactive exploration and analysis of metabolomics, transcriptomics, proteomics, and multi-omics datasets. Organized as a hierarchy of cellular systems, the Dashboard at its highest level contains graphical panels for the full range of cellular systems, including biosynthesis, energy metabolism, and response to stimulus. Thus, the Dashboard top level surveys the state of the cell across a broad range of key systems in a single screen. Each Dashboard panel contains a series of X–Y plots depicting the aggregated omics data values relevant to different subsystems of that panel, e.g., subsystems within the biosynthesis panel include amino acid biosynthesis, carbohydrate biosynthesis and cofactor biosynthesis. Users can interactively drill down to focus in on successively lower-level subsystems of interest. In this article, we present for the first time the metabolomics analysis capabilities of the Omics Dashboard, along with significant new extensions to better accommodate metabolomics datasets, enable analysis and visualization of multi-omics datasets, and provide new data-filtering options.

## 1. Introduction

In recent years, the exponential growth of high-throughput technologies has generated vast amounts of data, providing new insights into the molecular mechanisms governing cellular processes. Metabolomics, alongside other omics disciplines, has emerged as a powerful tool for comprehensively profiling the small molecules within a biological system. The integration of metabolomics with transcriptomics, proteomics, and other omics datasets offers the promise of a holistic perspective on the complex interactions within biological systems. At the same time, this flood of data presents real challenges to understanding, underscoring the need for sophisticated new analytical tools capable of extracting meaningful information from diverse datasets.

We have previously introduced the Omics Dashboard capabilities for interactive exploration and analysis of gene expression datasets [1]. The Omics Dashboard is unique in presenting the user with a high-level visual summary of a transcriptomics dataset in the context of an array of cellular systems to enable a scientist to quickly ascertain the state of those systems, identify systems of particular interest, and drill down to examine the detailed behavior of those systems. The Omics Dashboard was developed by the authors as a component of the BioCyc.org [2] website and the Pathway Tools software [3], version 27.5. Both systems provide a suite of metabolomics analysis tools [4] that include the abilities to paint metabolomics data onto individual pathway diagrams and onto zoomable whole metabolic network diagrams; metabolite enrichment analysis; calculating metabolic pathway activation scores from metabolomics data; calculating pathway covering analysis [5]; and searches of our metabolic databases by metabolite name, molecular weight, and chemical formula.

In this article, we present for the first time the metabolomics analysis capabilities of the Omics Dashboard along with a number of new developments including new multi-omics data analysis tools, extensions to its processing of metabolomics data, new data-filtering options, a new search capability, and a new data-table tool.

The Dashboard is structured as a hierarchy of cellular systems. At its topmost level it contains panels for cellular systems such as biosynthesis, energy metabolism, and non-metabolic functions. Each of these panels contains a series of X–Y plots depicting aggregated omics data values for genes, proteins, and/or metabolites within the subsystems of that panel. For example, the Biosynthesis panel includes individual plots for biosynthesis of amino acids, nucleotides, carbohydrates and others. As the user focuses on subsystems of interest, additional visualizations become available, such as pathway diagrams painted with omics data, and diagrams showing the operon organization of all genes within a given biological system. The Dashboard can accommodate metabolomics, transcriptomics, proteomics, and reaction-flux data (e.g., results of executing metabolic models), or any combination of up to three of the above datatypes. When multi-omics data are provided to the Omics Dashboard, all data modalities for a given system are displayed side by side and can be visualized as a cohesive unit.

By starting from a comprehensive overall survey of major biological systems, the Omics Dashboard can greatly reduce the time and expertise needed to understand complex datasets. An investigator can quickly observe and analyze the functioning of the entire metabolic system prior to analyzing the behavior of individual pathways. Once a system of interest is selected, the nested dashboard panels allow the user to drill down into successive levels of functional detail, and then pop back out to examine a different system. Thus, the investigator can rapidly explore many areas of cellular function at multiple levels of detail, all from a single screen. The knowledge of the high-level functional state of the organism can be applied as the investigator analyzes the details of the data, leading to greater insights.

### Related Work

We view the Omics Dashboard as complementary to the range of existing methods and tools available for analysis of metabolomics and multi-omics data. Other popular online tools for these kinds of analyses include MetaboAnalyst [6] and XCMS Online [7]. These platforms have the advantage of being able to import and process LC/MS spectral data directly, identify metabolites, and apply a variety of statistical univariate and multivariate analyses such as ANOVA, fold-change analysis, principal component analysis and partial least squares-discriminant analysis. In contrast, the Omics Dashboard will only process input files in which the metabolites have already been identified and any desired statistical analyses to generate fold changes or significance values have already been performed. Both MetaboAnalyst and XCMS Online can map metabolites to pathways, perform enrichment analyses, and display the results as plots or tables of various kinds that make it easy to identify the most significant pathways, and both can incorporate transcriptomics or proteomics data into these analyses and visualizations to varying degrees. MetaboAnalyst can also display pathway diagrams highlighted with significant genes and metabolites. However, neither of these platforms offers an overall high-level survey of all metabolic and other cellular functions in the context of an omics dataset, and their pathway plots and diagrams lack the information density and versatility of the Omics Dashboard panels. For example, their pathway plots are largely based on enrichment scores and counts of the number of metabolites associated with different pathways; they cannot show the details of a time-course experiment beyond whether a given metabolite is generally increasing or decreasing. By contrast, our displays show the entire shape of the experimental response over all timepoints, illustrate both individual metabolite values and aggregated values in a single plot, and allow metabolomics data to be visualized side by side with other omics data, all at multiple levels of detail.

## 2. Materials and Methods

The Omics Dashboard is a component of the Pathway Tools software [3] (new features described in this paper are available as of version 27.5). Pathway Tools powers the BioCyc website and is used to construct the organism-specific databases, called Pathway/Genome Databases (PGDBs), that make up the BioCyc database collection. The panel and plot visualizations within the Dashboard are implemented in JavaScript using Google Charts [8], version 52. The Dashboard also contains client-side (web browser) components implemented in JavaScript, and server-side components implemented in Common Lisp. The pathway diagrams displayed by the Dashboard are generated using Pathway Tools pathway collages (also developed by the authors) [9], which in turn are implemented using Cytoscape.js [10] version 3.9.4. The pathway collage software was extended as part of this work to better integrate with the Dashboard and to support the display of metabolomics and multi-omics datasets. The operon diagrams displayed by the Dashboard are generated by existing Pathway Tools algorithms.

The example data for all figures in this paper are from two publicly available datasets. Metabolomics example data were taken from Metabolomics Workbench study ST000016 by McDonnell et al. [11], which explores the metabolic effects of ALK inhibitor CEP-26939 on a human cell line that expresses the NPM-ALK oncogene for anaplastic large-cell lymphoma. For our figures, we used positive ion metabolite levels for CEP-26939 vs. DMSO. Replicates were averaged, and log fold-change values computed. Multi-omics example data came from a study by Sun et al. [12], containing metabolite and gene profiles over a 48-h time-course for fed-batch fermentation of an *Escherichia coli* strain engineered for L-phenylalanine overproduction.

## 3. Results and Discussion

### 3.1. Visualization of Metabolomics Data on the Omics Dashboard

**Figure 1:**
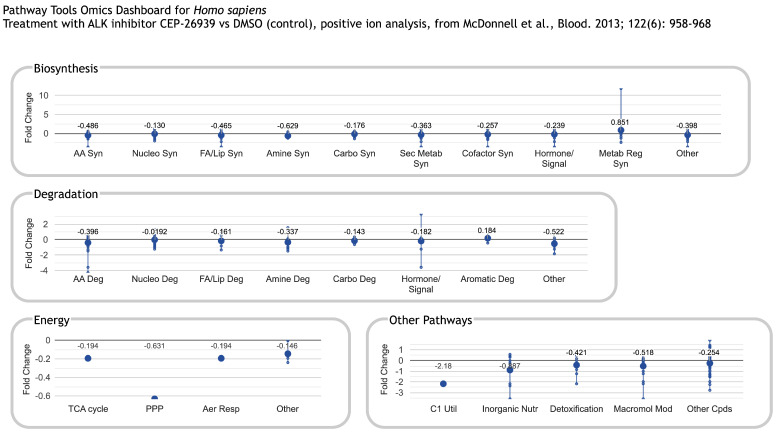
Omics Dashboard with metabolomics data from McDonnell et al. [11], exploring the metabolic effects of ALK inhibitor CEP-26939 on a human cell line that expresses the NPM-ALK oncogene for anaplastic large-cell lymphoma. The numbers for each subsystem (as well as the large dots on each line) indicate the average log fold change vs. control for all metabolites involved in that subsystem (except for some common metabolites, such as NAD+ or AMP, which have been excluded using our Data-Filtering module). The authors were not involved in the generation or analysis of this publicly available dataset; it is included here to illustrate Omics Dashboard capabilities.

The Omics Dashboard is organized into a set of panels representing top-level cellular systems, each consisting of a set of plots representing component subsystems (Figure 1). Each system and subsystem of the Omics Dashboard maps to one or more pathways or pathway classes, Gene Ontology (GO) terms, or computed categories based on transport or regulatory activity. The systems that are relevant for metabolomics analysis are all pathway-based, deriving from the MetaCyc pathway taxonomy. We group all of metabolism into four top-level systems (the dashboard panels): Biosynthesis, Degradation, Energy Metabolism, and Other Pathways. Each top-level system is divided into subsystems that correspond to MetaCyc pathway classes; for example Biosynthesis includes Amino Acid Biosynthesis, Nucleoside and Nucleotide Biosynthesis, Carbohydrate Biosynthesis, Secondary Metabolite Biosynthesis, and others. Amino Acid Biosynthesis is further divided into subsystems for each individual amino acid. At the lowest level are subsystems for individual pathways. Although derived from it, the Omics Dashboard systems and subsystems are not an exact mirror of the MetaCyc pathway taxonomy. Rather, we hand-selected which pathway classes should be explicitly included, and which should be combined into subsystems named “Other”, and flattened portions of the class hierarchy to limit the number of levels in the Dashboard hierarchy. This dataset shown in Figure 1 consists of a single treatment vs. control condition. The Dashboard is also capable of displaying datasets with multiple conditions or timepoints.

The set of subsystem plots visible within each panel is both organism-dependent and dataset-dependent. For example, in a PGDB for a photosynthetic organism (which contains photosynthetic pathways) the Energy Metabolism panel will include a Photosynthesis plot, but only if the uploaded dataset includes metabolites that participate in some photosynthetic pathway. For humans, plots for biosynthesis and degradation of hormones and other signaling molecules will automatically appear in the Biosynthesis and Degradation panels, respectively, if metabolites involved in those pathways are present in the dataset. The Dashboard for every organism uses the same set of pathway-class-based systems (although a class may be omitted if an organism has no pathways in that class), but the lowest level, or “base” subsystems, based on individual pathways, will vary from one organism to another, depending on the pathways present in that organism. The set of panels and plots is user-customizable: Pathways, pathway classes or GO terms can be added or removed to any existing panel, or a new top-level panel can be created with a custom list of pathways, pathway classes, genes or GO terms.

**Figure 2:**
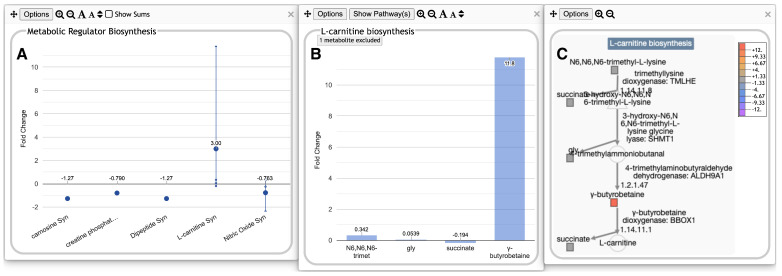
Omics Dashboard showing multiple levels of drill-down, using the same dataset from Figure 1. (**A**) Clicking on the “Metab Reg Syn” plot within the top-level Biosynthesis panel in Figure 1 pops up a panel for Metabolic Regulator Biosynthesis, with plots for individual regulator molecules. These plots use the same graphical conventions as for the top-level Dashboard in Figure 1. (**B**) Clicking on the “L-carnitine Syn” plot in (**A**) produces a “base panel” showing the individual omics data values for metabolites involved in this pathway. A message near the top of this panel indicates that one metabolite has been excluded due to data filtering (mousing over this message identifies the excluded metabolite as NAD+, not shown). (**C**) Clicking on the “Show Pathway(s)” button at the top of (**B**) produces a popup showing the pathway diagram overlaid with colored boxes indicating omics data values for all metabolites in the pathway.

The user can drill down into a given subsystem by clicking on the plot to reveal more details. For example, in Figure 1, the eye is immediately drawn to subsystems where one or more metabolites have a large fold change. Clicking on one of these, the Metabolic Regulator Biosynthesis plot (abbreviated as “Metab Reg Syn”—mousing over any plot will show a tooltip with the full subsystem name) within the Biosynthesis panel, produces a new panel with plots summarizing the biosynthesis of a single metabolic regulator (Figure 2A). Continuing to drill down on subsystems of interest will eventually produce a plot of all metabolites within a base subsystem (a subsystem that contains no subsystems) such as a metabolic pathway. For example, clicking on the L-carnitine biosynthesis plot in Figure 2A plots the metabolite changes for all metabolites in the dataset that participate in that pathway (Figure 2B).

**Figure 3:**
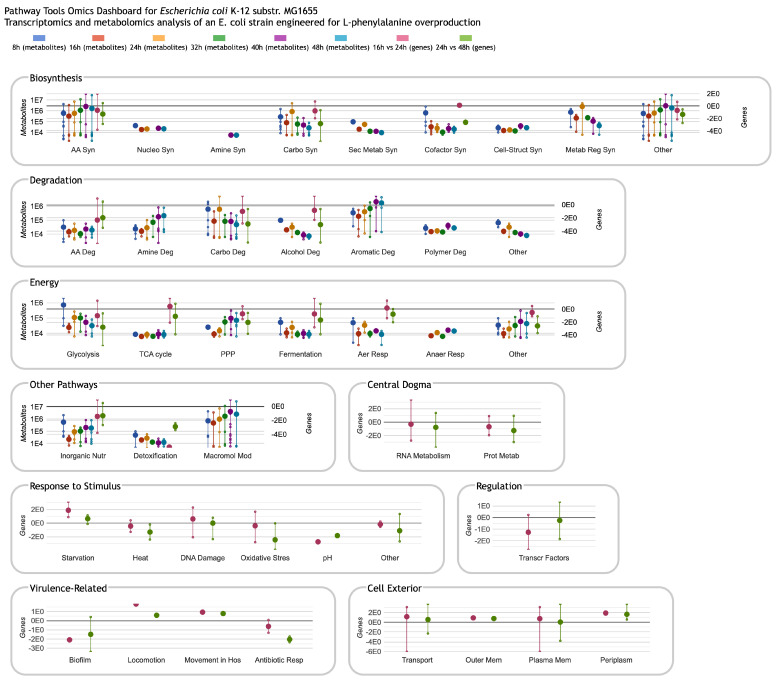
Omics Dashboard showing metabolite and gene profiling over a 48-h time-course from Sun et al. [12] of fed-batch fermentation of an *Escherichia coli* strain engineered for L-phenylalanine overproduction. The initial stages of fermentation are dominated by growth, with biomass reaching a maximum at 26 h. L-phenylalanine production begins at around 12 h and continues thereafter. The first six timepoints are normalized metabolite abundances at 8, 16, 24, 32, 40 and 48 h, respectively, and use the scale on the left side of each panel. The last two timepoints are transcriptomics log fold-change values (significant genes only) for 16 vs. 24 h (the growth period), and 24 vs. 48 h (the steady state period), and use the scale at the right of each panel. Because the published transcriptomics data includes significant genes only, several plots lack gene data, so show only the six metabolomics data columns (or even fewer if metabolites in the plot are missing data for some timepoints). The non-metabolic panels at the bottom do not include any metabolomics data, so show only the two transcriptomics data columns (and include only a single scale, at left). The authors were not involved in the generation or analysis of this publicly available dataset; it is included here to illustrate Omics Dashboard capabilities.

**Figure 4:**
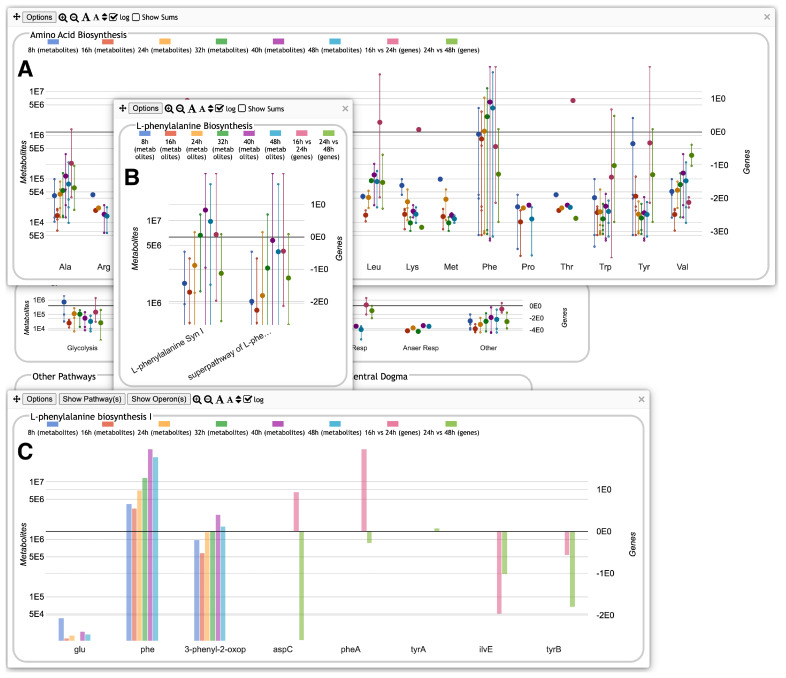
Omics Dashboard showing multiple levels of drill-down, using the same dataset from Figure 3. (**A**) Clicking on the “AA Biosyn” plot within the top-level Biosynthesis panel in Figure 3 pops up a panel for Amino Acid Biosynthesis, with plots for each individual amino acid. These plots use the same graphical conventions as for the top-level Dashboard in Figure 3. (**B**) Clicking on the “Phe” plot in (**A**) pops up a detail panel with plots for two different phenylalanine biosynthesis pathways. (**C**) Clicking on the first pathway plot in (**B**) produces a “base panel” showing the individual omics data values for metabolites (the first three bar graphs) and genes (the rest of the bar graphs) involved in this pathway.

The lines and dots within a plot for Figure 1 and Figure 2A can be interpreted as follows: Each vertical line represents a single data column from the input dataset, such as a single timepoint or experimental condition. The dataset shown in Figure 1 and Figure 2 contain only one data column, so there is one vertical line per subsystem. If there are multiple timepoints or conditions, each is drawn using a different color, and a legend mapping colors to data columns is included at the top of the main display as well at the top of each popup panel, as seen in Figure 3 and Figure 4.

Each small point shown along a vertical line corresponds to a data value for a single metabolite associated with the cellular subsystem represented by that plot. The larger circle along each line is the average of all data values for that subsystem. When there is only one data column, the average is also displayed as text above the larger circle; this text is suppressed when multiple data columns are present. Each panel uses its own Y-value scale, initially determined by the data values represented in that panel, but user-customizable. For many kinds of data, it may be more informative to plot the values on a log scale. This is a preference that can be set either on a per-panel basis or as a global default (this option is not available for log fold-change data, in which the data values are already log values). Other controls let the user change panel size, font size, and order (both panels themselves and of plots or metabolites within a panel).

When the software generates a Dashboard subsystem based on pathways for a given organism, database queries are issued to determine which pathways are defined within that organism, and what are the full set of metabolites associated with those pathways. The software then identifies which of those objects are present in the supplied dataset(s). This may require traversing the MetaCyc compound class hierarchy. For example, one of the metabolites in the TCA cycle is D-*threo*-isocitrate. However, a metabolomics dataset based on an experimental procedure that is unable to distinguish stereoisomers may report simply isocitrate. The software must recognize that isocitrate includes all its stereoisomers and associate the isocitrate data values with all subsystems that include the TCA cycle or any other pathways that involve any isomer of isocitrate.

The combination of all these elements produces an extremely powerful, information-dense display. Looking at a single plot, it is possible to perceive at a glance how the range, distribution and average of components of that subsystem all change over the course of an experiment, and how the distribution for one subsystem compares to that of other related subsystems in the same panel. The eye is naturally drawn to subsystems that show interesting trends, and the intuitive user interface makes it easy to focus on subsystems that may merit further study.

For the base subsystems representing a single pathway such as L-carnitine biosynthesis, the panel produced is a simple graph of the omics values for all metabolites associated with the pathway across all timepoints or data columns. At this level, the panel header includes an additional button to display the pathway diagram overlaid with omics data, as shown in Figure 2C. To make it easy to obtain more information, these displays all include copious hyperlinks to show the corresponding BioCyc pages in a new tab.

### 3.2. Support for Multi-Omics Datasets

A new capability of the Omics Dashboard is the ability to support multi-omics datasets with either two or three component datasets. The different component datasets are processed separately and can use different scales, but are displayed side by side together in the same plots. As an example, Figure 3 shows a multi-omics dataset that combines metabolomics and transcriptomics profiles. The first six colors in the legend at top correspond to metabolomics timepoints and map to the first six vertical lines and dots in each plot in the first four panels, such as the AA Syn plot in the upper left (in some subsystems, there is no data for one or more timepoints; in that case the corresponding colored lines and dots are omitted). The last two colors in the legend represent transcriptomics fold changes (log ratios) for two different timepoint intervals and appear as the last two sets of lines and points for every plot for which these data are available. The lines representing metabolomics columns include dots for individual metabolite values, whereas the lines representing transcriptomics columns consist of dots for gene values. Thus, all gene and metabolite data for a given subsystem are visualized together as an integrated unit. Figure 4 shows the displays generated when focusing on amino acid biosynthesis and then on phenylalanine biosynthesis. The base panel, Figure 4C, now shows both metabolites and genes associated with a pathway.

Multi-omics panels employ a dual Y-axis, with the first scale indicated on the left side of each panel and the second scale on the right side. In the example of Figure 3 and Figure 4, the lines and dots in the first six colors in every plot (representing metabolite quantities) use the scale at left, and the last two colors, representing gene expression fold changes, use the scale at right. To make it easier to tell which colored bar uses which scale, by default the omics datatype is included within the legend text for each timepoint, and the axis labels indicate which omics datatype(s) they apply to. Users can edit both the axis labels and the data column legend text, but it is recommended that they retain enough information for viewers to match each data column to its corresponding axis. The Omics Dashboard can display multi-omics datasets consisting of either two or three component datasets. If there are two component datasets, each will have its own dedicated Y-axis, but if there are three component datasets (e.g., metabolomics, transcriptomics and proteomics), then two of them must share one of the Y-axes. In some cases, the choice of which must share is constrained: if two of the datasets contain log fold-change data and the other contains absolute quantities, or vice versa, then the two with the same type of data must share a Y-axis. If all three datasets have compatible data, then the choice is arbitrary and the user can change it at any time. The user can also specify which Y-axis appears on the left side and which on the right side of each panel, and can assign different colors for the grid lines of each scale.

**Figure 5:**
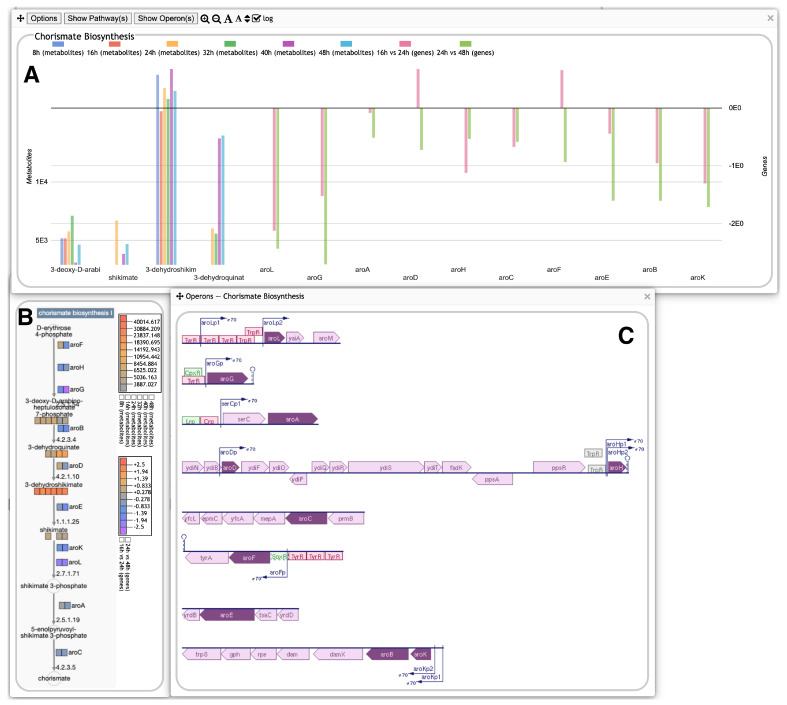
Omics Dashboard showing three different detail views of the chorismate biosynthesis pathway using the same dataset from Figure 3. (**A**) A graph of the metabolite and gene expression values for the metabolites and genes in the pathway. (**B**) The pathway diagram, overlaid with metabolite and gene expression data as colored boxes. Different color scales, shown to the right of the diagram, are used for metabolomics vs. transcriptomics data. (**C**) Operon diagrams for all genes involved in the pathway.

The base panels again include a button to show the pathway diagram, overlaid now with both metabolomics and transcriptomics data, each with its own color scale, as shown in Figure 5. An additional button will display operon diagrams for all pathway genes in the dataset.

The Data Column Selection control panel, in addition to selectively hiding or showing individual columns, also lets the user reorder the columns. By default, multi-omics data are ordered by component dataset, e.g., all data columns from the first dataset, then all from the second dataset, then all from the third. However, if each dataset includes columns for the same sets of conditions or timepoints, the user might prefer to see all metabolomics, transcriptomics and proteomics data for condition 1, followed by all data for condition 2, and so forth. The Dashboard controls make it easy to drag column labels to reorder the columns in the display. This control panel also allows the user to update the colors used for each data column, as well as the labels.

One advantage of the Omics Dashboard over other metabolomics platforms that incorporate multi-omics data is that the Dashboard includes several panels for non-metabolic cellular functions. These non-metabolic subsystems, for use with transcriptomics or proteomics data, are derived from GO term assignments. As for pathway classes, we do not attempt to mirror the entire GO taxonomy. Rather, we have hand-selected nearly a hundred GO terms that we consider to be of general interest, and grouped them into top-level systems for Central Dogma (including transcription, translation, and related functions), Response to Stimulus, Cellular Processes, Virulence-Related and Cell Exterior. To populate these systems and subsystems for a given organism, a query is issued against the database to find all genes or proteins annotated to the corresponding GO terms or any of their child terms. Some additional subsystems are generated based on the transport or regulatory activities of gene products as represented in the database without using GO terms. Although metabolomics data cannot be displayed on these panels, when both metabolomics and either transcriptomics or proteomics data are provided, the non-metabolic panels will be displayed as if the input were a single-omics transcriptomics or proteomics dataset. This makes it possible to see both metabolic and non-metabolic responses to experimental conditions on a single screen. For example, it can be informative to observe how changes in metabolite values might align with, say, a stress response or transporter expression.

### 3.3. Data Filtering

Another new extension to the Omics Dashboard is the incorporation of multiple customizable filtering options. When datasets are large, it can sometimes be difficult to visually pick out interesting trends against the much larger backdrop of entities that do not change very much. By filtering the data to show only the most relevant items, it becomes easier to identify the overall effects of an experiment. In the past, if users wanted to view a filtered subset of their data they would have to prepare a separate datafile containing only the data values that meet the desired criteria. With such functionality now incorporated directly into the Omics Dashboard it becomes simple for a user to try out different combinations of filters and thresholds to obtain the most value from their dataset. Filters are applied on a per-entity basis; if a gene, metabolite or protein meets the filtering criteria for any visible data column, then all its data columns are included in the various graphs, even those that do not meet the filtering criteria. For example, if a data value filter is applied to a dataset with two data columns, it could be that one of the data column values for a given metabolite exceeds the filter threshold value but the other does not. Our displays will include all the data for this metabolite, not just the one data value that exceeds the threshold. This is for two reasons: (1) once an entity is identified as being of interest (because it passed the filtering criteria at least once), trends in the data become more apparent if all values are shown, and (2) the computing of averages and the displays showing the distribution of data values for the higher-level subsystems is really only meaningful if the same sets of entities are being considered across all timepoints or conditions.

The following filters are available:

- **Filter by Data Value.** Users specify either a minimum or maximum threshold value, and only entities that have a data value either above or below the threshold for at least one of the visible data columns will be included in the display. Filtering can be based on either the actual data value or the absolute value of the data value. This latter capability is especially useful if the values represent fold changes and the user is primarily interested in entities that have changed a lot, either in the positive or negative direction.
- **Filter by Significance Value.** Many datasets will include additional columns of statistical significance values; for example, *p*-values derived from replicate analysis or signal-to-noise ratios derived from batch effect correction algorithms (the Omics Dashboard does not have the ability to compute such values; all normalization and significance calculations must be performed prior to uploading data to the Dashboard). Although, in general, the user will not wish to display these significance values in the various panels, they can be used for filtering. For each visible data column the user designates a corresponding significance column. An overall significance threshold is also specified, and whether that threshold is a maximum or a minimum; for *p*-values, this will be a *p*-value maximum. For an entity to pass the significance filter, its significance value for at least one visible data column must match the threshold criteria. It is not necessary for every data column to have a corresponding significance column, but only columns that do will be considered for significance filtering.
- **Exclude Common Metabolites.** This filter is available only for metabolomics datasets or multi-omics datasets that include a metabolomics component. Visualization of metabolomics data in the Omics Dashboard presents special challenges because some metabolites are present in many metabolic pathways (e.g., ATP, NADP, L-glutamate) and therefore are not accurate indicators for the activity level of a specific pathway. Their presence in the Dashboard can have a distorting effect and hinder the user’s assessment of pathway activity levels. To mitigate this problem, we present to the user a checklist of metabolites from the input dataset that participate in ten or more pathways (this threshold is customizable). The user can then select any that should be excluded from most systems. The relevance of a given metabolite is system-dependent, however. For example, since L-glutamate participates in so many different pathways, suppressing its display in most pathways in which it participates will likely make it easier to see other, more informative metabolite changes in those pathways. But levels of L-glutamate are highly relevant for the pathways of glutamate biosynthesis and degradation for which L-glutamate is the primary input or output, so we do not want to suppress its display for those subsystems. Fortunately, for many (but not all) pathways in MetaCyc, a curator has designated certain metabolites as primary inputs or outputs. Thus, even if a common metabolite has been selected for exclusion, we can still show it in pathways for which it has been designated a primary input or output, as well as all systems and subsystems that include those pathways.

If multiple filtering criteria are specified, then an entity must meet all of them to be included in the displays. For the base panels, in which graphs for each individual entity are shown, if there any additional entities that have been filtered out, the number of excluded entities will be indicated in a message near the top of the panel (see Figure 2B)—a tooltip for this message lists the excluded entities.

### 3.4. Other Dashboard Capabilities

Several other Dashboard capabilities have been described previously [1]. Three newer features merit mention here.

**Figure 6:**
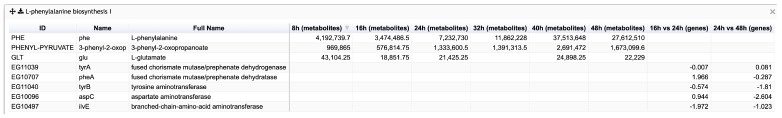
A data table generated from the base panel for phenylalanine biosynthesis from Figure 4C, containing both metabolites and genes and their data from the relevant data columns.

- A Search option lets the user type in the name of any entity (with auto-completion) to search for all base panels that include that entity. The user can then select one to show it.
- The Options menu for any panel includes a command to Show Data as Table. A table is generated containing all the data for just that subsystem, which can be viewed or downloaded. An example for a base subsystem is shown in Figure 6. For a multi-omics dataset, this table will include rows for all entities (genes, metabolites and/or proteins) and columns for all data columns. The table can be sorted by any column by clicking on the column header.
- For any dataset, there may be one or more entities that do not appear in any Omics Dashboard subsystems, for example, a metabolite that is not a participant in any defined pathway, or a gene that is neither an enzyme nor annotated to any of the GO terms that make up the Dashboard. If such entities exist, a button labeled “Show Objects Not Present in any Subsystem” will appear below all other Dashboard panels, which will bring up a base panel with individual graphs for all such entities.

### 3.5. Invoking the Omics Dashboard

The Omics Dashboard is one of many tools available at the BioCyc.org website. Users can also install the Pathway Tools software at their own site to use the Omics Dashboard on their own organism databases.

To access the Omics Dashboard from BioCyc, go to https://biocyc.org/ (accessed on 16 January 2024), select the organism of interest using the “Change Current Database” button, and invoke the command Tools→Analysis→Omics Dashboard. In the resulting entry form, indicate whether you are supplying single-omics or multi-omics data. Data can be uploaded from a tab-delimited text file in which the first column contains metabolite, gene or protein names or identifiers, and one or more subsequent columns contain the relevant data values. Alternatively, data can be uploaded one time to a Pathway Tools SmartTable [13] and then imported from the SmartTable to the Omics Dashboard. When specifying a data source, the user must indicate the type of data in the first column (e.g., metabolites for metabolomics data, genes for transcriptomics data), which additional columns contain the data values the user wishes to analyze, and whether the data are absolute values (e.g., counts or intensities) or relative (e.g., ratios or log ratios). When uploading multi-omics data, the user will need to specify multiple (two or three) files or SmartTables, one for each datatype, each with its own set of parameters. Alternatively, a single combined file can be created that contains all the datasets plus a metadata section with all required parameters. The file formats are described in more detail in the User Guide [14].

We recommend that normalization and significance calculations be applied before importing data into the dashboard. Columns of significance values can be uploaded alongside other data columns—in general the user will wish to hide these in the main display, but the Dashboard can use them for significance filtering. If there are multiple replicates for each timepoint or condition, we also recommend averaging them before upload, although this is not required (except for multi-omics data). Although the Omics Dashboard can average groups of replicates for you, overall performance may be adversely affected. Replicate averaging is not supported in multi-omics mode.

## 4. Conclusions

The Omics Dashboard adds a new dimension to the analysis and interpretation of omics data. The top-level hierarchical approach based on systems of cellular function enable the investigator to gauge the relative activity levels of different cellular systems and quickly identify subsystems of interest. Unique, powerful visualizations can lead to new insights and enhance presentations and publications. The new extensions described here greatly increase the utility of the Omics Dashboard for the analysis of metabolomics and multi-omics datasets. In particular, the ability to view metabolomics results side by side with transcriptomics or proteomics data facilitates rapid understanding of these complex systems.

## Data Availability

The Omics Dashboard is freely available in conjunction with the EcoCyc *E. coli* database at BioCyc.org; a paid subscription is required for its use with other of the 20,000 BioCyc databases. The Omics Dashboard is freely available for academic research purposes in conjunction with the Pathway Tools software [15]; a fee applies to other types of use. Source code is available upon request. No new data were created or analyzed in this study.

## Abbreviations

The following abbreviations are used in this manuscript:

PGDB	Pathway Genome Database
GO	Gene Ontology
